# Supplementary material for: Management of veterinary anaesthesia in small animals: A survey of current practice in Quebec
Source: PLoS One. 2020 Jan 16;15(1):e0227204. doi: 10.1371/journal.pone.0227204 (PMC6964820; doi:10.1371/journal.pone.0227204)
Supplement: Attachment — Submitted filename: Responses to the Reviewer_13 novembre 2019.docx [file pone.0227204.s005.docx]

**Responses to the Reviewers’ comments on the manuscript**

**Title: Management of veterinary anaesthesia: A survey of current practice in Quebec**

**Ref:** PONE-D-19-16258

**Journal:** PLOS ONE

**Date:** 2019-11-10

______________________________________________________________________________

The authors would like to thank the Editorial Board and Reviewers for careful review of our manuscript and providing us with the comments to improve its quality. We have judiciously taken them into consideration in preparing our revision. The following detailed responses have been prepared to address the reviewer’s comments.

***Reviewer #1***

______________________________________________________________________________

**General comment:**

*Thank you for the opportunity to review this interesting manuscript. As a whole, I think it can provide interesting insight into the practice of small animal anaesthesia however I think the authors need to better explain why they feel that practice in their subsect (French Eastern Canada) might differ from elsewhere and, hence, why the study was conducted. In doing this, the end of the discussion also needs a bit better of a conclusion about what the reader should do with this information. The authors need to provide some statement about what comes next or how to fix the problem. In addition, as this was only a study of anaesthesia practice in small animal settings, I believe that needs to be made more clear. I suggest adding "small animal" to the title and including it at various places throughout the text body as well. The is critical as anaesthesia in equine, ruminant, exotic or zoo practice might differ profoundly. Also, I have some concerns about whether this study was powered sufficiently, given the relatively low survey response rate, to elucidate differences if they existed. No mention of a power study was included. Finally, I've done some minor editing however I would recommend that the manuscript be reviewed for English grammar*

*as some of the wording is awkward.*

*As the original survey and subsequent data included in the supplementary attachments was in French, I was unable to review them for validity and appropriate representation within this manuscript.*

**Response:**

The authors thank the Reviewer for his/her general comment and rigorous revision about the present manuscript and the recognition that the main subject is of interest. First, the remark of thee Reviewer #1 is very judicious. Quebec Province is the only one to be French speaking and culture in the whole North-American continent. The Province houses the only French-speaking Faculty of veterinary medicine in North-America, fully-accredited by the American Veterinary Medical Association and the Canadian Veterinary Medical Association. Therefore, the (small animals) veterinary practitioners community of Quebec looked to us attractive to survey with regards to its expected homogeneity (favored by its cultural exception) and how it integrates the internationally-adopted standards in small animals anaesthesia. Indeed, as indicated in the Introduction (Lines 50-51), “a previous study showed some geographical heterogeneity between Canadian provinces in analgesia management [17, previously 6]”. Therefore, we were curious to know the situation for small animals anaesthesia. After this, our goal is to compare it to the Rest of Canada situation. Secondly, as suggested, we have added the precision of the field "small animals" in the title and throughout the main text. Moreover, we added a conclusion highlighting more important aspects of the survey results, hypothesis of explanation for the results (*what the reader should do with this information*). In addition, we provided the next step (*statement about what comes next*) and *how to fix the problem* (Lines 565-585).

Considering the potential number of responses that could be collected, the response rate to the questionnaire used to collect the data was **20.85 %** (156 respondents out of 748 sent invitations to small animals practitioners). However, a total of 210 veterinarians visited the questionnaire webpage and 156 of them provided a comprehensive set of responses for analysis, representing a response rate of 74% among those who showed interest. Although the first rate may appear to be low at first glance, it is well recognized that, on average, a rate of **11%** is usually obtained in external surveys [1]. The response rate in this study is therefore significantly above average (almost double). The potential bias caused by the non-response rate cannot be ignored, but it doesn’t make it possible to judge the quality of the representativeness of the data collected [2].

Finally, we made many other changes following your comments for some areas of the manuscript, leading to a more concise and useful revised manuscript.

Minor point #1:

*Line 22: consisted OF four parts, not "in"*

**Response:**

We thank the Reviewer #1 to point out this error. We have made the correction in the revised manuscript Line 22.

Minor point #2:

*Line 24 and throughout the text: Year and Type do not need to be capitalised*

**Response:**

We have judiciously corrected these mistakes in the revised manuscript.

Minor point #3:

*Line 30: insert the word "prior" between evaluation and to*

**Response:**

Thank you, we have inserted the term suggested by the Reviewer #1 in the revised manuscript.

Minor point #4:

*Line 33: Avoid emotive language. Change to "Some practices are more compliant..."*

**Response:**

We have carefully considered this comment and made the change suggested by the Reviewer #1 (Line 33).

Minor point #5:

*Line 39: Insert "small animal"*

**Response:**

The insertion have been made in the revised manuscript. Thank you for the suggestion.

Minor point #6:

*Line 41: Lumb and Jones is an inappropriate reference for this statement. Please reference the original papers here (you reference them later anyhow) and move the reference to the end of the sentence after the sick patients as that is part of the reference.*

**Response:**

The Reviewer #1 is right, and we moved the original research references at this statement.

Minor point #7:

*Line 42: Using "should also" implies that you've previously stated something else to evaluate. Delete this.*

**Response:**

We have deleted these terms, as suggested by the Reviewer #1 (Line 42).

Minor point #8:

*Line 43: Again, more appropriate references should be used here (Hosgood and Scholl for example).*

**Response:**

Once again, the Reviewer #1 is right, we indicated the appropriate original references, and we kept the Lumb & Jones chapter with regards to its popularity in the field and the fact that the statement was generic, exactly referring to this chapter (Line 44).

Minor point #9:

*Line 45 and other places: The WSAVA and AAHA/AAFP guidelines do not specifically make best practice recommendations about anaesthesia so this is a bit misleading. Consider changing your discussion around these references to refer more specifically to peri-operative analgesia. Also, this should include Warne et al 2018 (AVJ 96(11)) in the references.*

**Response:**

The Reviewer #1 is right, and modifications have been brought to refer to “peri-operative analgesia” as well as to include Warne *et al*. 2018 as reference (Line 46).

Minor point #10:

*Line 47: Not sure what is meant by "nature of professional interaction"? This statement also probably should be referenced.*

**Response:**

We have changed “nature of professional interaction” for “professional environment” and included adequate reference (Lines 48-49).

Minor point #11:

*Line 62: Please describe the validation process (pilot survey with focus group?).*

**Response:**

We thought that the following section (Lines 60 to 63) was covering this point. However, while considering the Reviewer #1’s comment, we decided to modify it, and added some precisions: “The internal content and construct validation included a pilot survey with a focus group. The latter included various degrees of expertise in veterinary anaesthesia, from veterinary student, general practitioner to anaesthetist in private practice and academia. They evaluated and validated all sections as well as all used terminology to be perfectly understood for any registered veterinary general practitioner, which was the expected audience of the survey (Lines 61 to 65).”

Minor point #12:

*Line 65: Should read "fell under the Article"*

**Response:**

We have corrected the grammatical error (Line 67). Thank you.

Minor point #13:

*Table 2: "large team practices" is never defined*

**Response:**

The Reviewer #1 is right and we added the definition (5+ veterinarians) in Table 2 for “Large team practices”.

Minor point #14:

*Line 117: "on the subsequent responses"*

**Response:**

We have added the term "subsequent" in the revised manuscript (Line 120).

Minor point #15:

*Line 133: How were paediatric patients defined?*

**Response:**

The provided definition was **“**Paediatric patients are classically under 3 months old, or as you define them in your practice.”

Minor point #16:

*Line 169: Replace "easily" with commonly*

**Response:**

As suggested by the Reviewer #1, the adverb "easily" have been replaced with "commonly" (Line 172).

Minor point #17:

*Line 177: ASA Status needs a reference and it is never defined or explained anywhere prior to this point. Please add it to the introduction.*

**Response:**

This mention has been added in the Introduction (see Lines 42-44) with adequate references.

Minor point #18:

*Emergency drugs section: I would question whether monitoring status, monitoring BP in particular, had an influence on these results. Have you done any regression analysis on potential confounders?*

**Response:**

This is an excellent question. The Reviewer #1 was right. We found a relationship between monitoring of cardiovascular function, mostly heart rate and systemic arterial blood pressure, and use of anticholinergics and catecholamines-like substances. However, this was biased by an over-representation of respondents in referral centre, particularly for the use of vaso-/cardiotonic drugs. Therefore, we decided to not emphasize on this point.

Minor point #19:

*Lines 212 and others: Reporting in the negative is odd and a bit hard for the reader to interpret. For instance, stating that fewer respondents who were more recent grads never use midazolam than older grads would make a bit more sense rewritten as "Veterinarians who graduated more than 15 years ago were more likely to report never using midazolam than those who graduated more recently." Please consider changing all instances of negative reporting for clarity.*

**Response:**

The Reviewer #1 is right, and the three sentences were inversed, as suggested (see Lines 216-221). This was also done in Lines 238-241, Lines 336-337, Lines 376-377, Lines 380-382 and Lines 401-403.

Minor point #20:

*Lines 214-216: As the survey is in French, I can't clarify but were the drug questions specifically about the premed period or could they have been interpreted as "at any time"? Also, was "routine surgeries" defined for participants? I ask because I work in referral practice and I would rarely use either midazolam or fentanyl in cases that I defined as "routine".*

**Response:**

This section, as indicated in the text, was referring to “routine” surgeries, and only for premedication, not “at any time”. Please, note that the mention was about “never using” the drug.

Minor point #21:

*Line 228 and others: Should read "referral centre more often use"....this displacement of the verb occurs multiple times within the text.*

**Response:**

We have modified the verb position over all the manuscript in the revised manuscript. We thank you the Reviewer #1 for this correction.

Minor point #22:

*Line 262: Please rewrite the line "all respondents use NSAIDs as, I would hope, it is misleading. Certainly they don't all use them on every case which is how I read this. Consider "All respondents use NSAIDs when appropriate" or something along those lines.*

**Response:**

We thank the Reviewer for pointing out this confusing sentence. We made the change as suggested (Line 266).

Minor point #23:

*Line 303: Please change the word "benefit" as this study did not evaluate outcomes.*

**Response:**

We have replace the term "benefit" by "undergo" (Line 307 and 309).

Minor point #24:

*Line 363: May need to change the end of this as many practices weren't monitoring ventilation so how would they "know if they needed it"?*

**Response:**

The Reviewer #1 is right. The sentence has been modified (Line 367-368) for “… if they suspect they would need it”.

Minor point #25:

*Lines 378-382: Should probably highlight more clearly this super low response rate somewhere in the discussion.*

**Response:**

The Reviewer #1 is right, and this has been highlighted in the beginning of the Discussion (Lines 422-424).

Minor point #26:

*Line 407: Need to provide references here again.*

**Response:**

As judiciously requested by the Reviewer #1, this has been completed (Line 411).

Minor point #27:

*Line 425: Please change to "The evaluation and preparation of the patient appears to be sub-optimal in many instances in this study."*

**Response:**

We made the replacement of the sentence in the revised manuscript, as requested (Lines 430-431).

Minor point #28:

*Line 429: Please rewrite "Situation is even worse for...." to avoid emotive language.*

**Response:**

We took good care to rewrite this sentence as requested by the Reviewer #1 while avoiding to include an emotive language (Lines 435-436). Thank you for the recommendation.

Minor point #29:

*Line 433: Please change reminding to mentioning.*

**Response:**

Thank you, we have made the change in the revised manuscript (Line 438).

Minor point #30:

*Entire fasting section: What about Savvas et al 2009, VAA 36(6)?*

**Response:**

Once again, this is a good point. Studies to examine the influence of fasting duration and food type on the occurrence of reflux determined that shorter pre-anaesthetic fasting did not significantly increase the likelihood of regurgitation, though gastric contents were greater [3]. Additionally, the composition of the food had some effect on gastric emptying and risk of anaesthetic regurgitation [4]. There are several factors influencing the presentation of reflux in anesthetized dogs, which may be inherent to the patient (e.g. age, sex, breed, **weight**, or body condition), **medications** used prior to and during anesthesia, **type of surgery** or **position** of the animal during surgery. The multiplicity of situations makes it too complex, in our mind, for being introduced in the survey.

Minor point #31:

*Line 437: Should read "Guidelines regarding provision of an informed consent are well followed." Delete the part from "and so" as this doesn't add anything to the discussion and you don't unpack it to consider why further.*

**Response:**

This point has been modified in the revised manuscript as suggested by the Reviewer #1, thank you (Line 442).

Minor point #32:

*Line 440: Missing the word "documented" before concerns.*

**Response:**

We thank the Reviewer #1 for his/her rigorous revision. Therefore, the term "documented" has been added in the revised manuscript (Line 444).

Minor point #33:

*Line 450-455: Flip the order of these sentences so that the initial first sentence doesn't read like an opinion sentence.*

**Response:**

We thanks the Reviewer his/her comment. We have effectively flip the sentence in the revised manuscript while changing the terms used to avoid opinion sentence (Lines 454-459).

Minor point #34:

*Line 458: Is this specific for post-op or any analgesia at all?*

**Response:**

It is variable with the concerned practices. More usually, it is referring to post-op analgesia, but for some practices, it is question about the “analgesic combo” to provide over the surgical procedure.

Minor point #35:

*Lines 462-465: Rewrite as "This is inadequate in light of the discovery of a very low expression....."*

**Response:**

The sentence has been rewritten as suggested by the Reviewer #1 in the revised manuscript (Lines 467-470).

Minor point #36:

*Lines 465-466: Add "the" before dog and rat.*

**Response:**

This has been added, thank you (Lines 468, 469).

Minor point #37:

*Line 467: Should be "why" not "that" and "effective" not "efficient"*

**Response:**

Both substitutions suggested by the Reviewer #1 were made in the revised manuscript (Lines 470-471).

Minor point #38:

*Line 470: Change proper to "sufficient"*

**Response:**

The change have been made (Line 474), thank you.

Minor point #39:

*Line 472: Change "If" to "While"*

**Response:**

The correction has been made (Line 475), thank you.

Minor point #40:

*Line 474: Consider adding a referenced statement about low relative risk with loco regional anaesthesia.*

**Response:**

This has been added to the text, as requested (Line 478).

Minor point #41:

*Line 486: I think more recent fluid guidelines would be more appropriate here. Consider 2013 AAHA/AAFP guidelines.*

**Response:**

We agree with the Reviewer #1 suggestion, there was a mistake in the associated reference and the reference has been replaced with those proposed (Line 490).

Minor point #42:

*Line 496: Define "severe" hypotension*

**Response:**

The information has been added (see Lines 499 to 503).

Minor point #43:

*Line 500: Is there necessary pain?*

**Response:**
The Reviewer #1 is right, and the text has been modified for “… surgically-induced pain” (Line 506).

Minor point #44:

*Line 510: Readily should be "ready"*

**Response:**
We have modified the word in the revised manuscript (Line 516), thank you.

Minor point #45:

*Lines 511-514: Please add data from Carter et al, 2017 JAAHA 53(4)*

**Response:**

We thank you the Reviewer #1 for suggesting this addition. A sentence has been added, referring to this publication (Lines 523-527).

Minor point #46:

*Line 518: Change reference to referral*

**Response:**

We have replace the term "reference" by "referral" (Line 528), thank you.

Minor point #47:

*Line 521: Change undergraduate to "veterinary" as many programmes are NOT undergraduate.*

**Response:**

We have made the change in the revised manuscript following Reviewer #1’s comment (Line 531), thank you.

Minor point #48:

*Lines 523: The demographic results revealed some interesting associations. Not surprisingly, a clear gender effect was associated with the year of.....*

**Response:**

We agree with the Reviewer #1’s suggestion and the text has been modified accordingly (Lines 532-534).

Minor point #49:

*Line 525: Add "This is consistent with current veterinary school enrolment data that shows that approximately 80% of students enrolled in veterinary school are female. (AAVMC Annual Report 2018)"*

**Response:**

We agree with the Reviewer #1’s suggestion, and the corresponding text has been added, with reference (Lines 534-536).

Minor point #50:

*Line 527: However, overall, year of graduation does not influence the standard of care reported in this study.*

**Response:**

We agree with the Reviewer #1’s suggestion, and the corresponding text has been changed (Lines 539-541).

Minor point #51:

*Line 535: What is meant by "formation"?*

**Response:**

In order to avoid confusion, we have replaced this term by "training". We're sorry, we did not use the right term (Line 546).

Minor point #52:

*Line 536-538: I highly doubt this study was powered sufficiently to make this claim.*

**Response:**

We thank the Reviewer for this comment. This point has already been discussed before. Please refer to the response addressed in the general comment of this document page 2. However, we understand the fear of the Reviewer #1 and the following text has been added: “… such as reflected in the survey (with all its limitations: voluntary response on an electronic survey, limited power of analysis on a localised population, …).” (Lines 550-551).

Minor point #53:

*Line 542: Remove "possibly worse"*

**Response:**

We have removed those words in the revised manuscript (Line 555), thanks for the comment.

***Editor***

__________________________________________________________

*Dear Dr Eric Troncy,*

*Thank you for submitting your manuscript to PLOS ONE. After careful consideration, we feel that it has merit but does not fully meet PLOS ONE’s publication criteria as it currently stands. Therefore, we invite you to submit a revised version of the manuscript that addresses the points raised during the review process.*

**Response:**

We thank the Editor about his general comment concerning our manuscript. As recommended by the Reviewer, the modifications have been carried out and the essential revisions have been clarified in the answers addressed for each point. Thank you.

**References**

1.	Poole A. Michigan State University ed, editor. What is an acceptable survey rate? National Social Norms Center 2014. online.

2.	Johnson TP, Wislar JS. Responses rates and nonresponse errors in surveys. J Am Med Assoc. 2012; 307(17):1805–1806. https://doi.org/[10.1001/jama.2012.3532](https://doi.org/10.1001/jama.2012.3532)

3.	Savas I, Raptopoulos D. Incidence of gastro-oesophageal reflux during anaesthesia, following two different fasting times in dogs. Vet Anaesth Analg. 2000; 27(1):59–60. <https://doi.org/10.1046/j.1467-2995.2000.00008-11.x>

4.	Savvas I, Rallis T, Raptopoulos D. The effect of pre-anaesthetic fasting time and type of food on gastric content volume and acidity in dogs. Vet Anaesth Analg. 2009; 36(6):539–546. <https://doi.org/10.1111/j.1467-2995.2009.00495.x>
